# A Red List of mites from the suborder Uropodina (Acari: Parasitiformes) in Poland

**DOI:** 10.1007/s10493-018-0284-5

**Published:** 2018-08-23

**Authors:** Agnieszka Napierała, Zofia Książkiewicz-Parulska, Jerzy Błoszyk

**Affiliations:** 1 Department of General Zoology, Faculty of Biology, AMU, ul. Umultowska 89, 61-614 Poznan, Poland; 2 Natural History Collections, Faculty of Biology, AMU, ul. Umultowska 89, 61-614 Poznan, Poland

**Keywords:** Invertebrates, Arthropods, Soil fauna, Threatened species, Extinction, IUCN

## Abstract

This article presents a Red List of mite species from the suborder Uropodina (Acari: Parasitiformes) occurring in Poland. Evaluation of the conservation status of the analyzed species was compiled on the basis of new criteria, which may also be applied to other groups of soil fauna. The authors employ the names of categories proposed by the International Union for Conservation of Nature (IUCN). One of our aims was to review the IUCN criteria to ascertain whether they are applicable in an attempt to assess the danger of extinction of soil invertebrates, and to see whether the criteria can be adapted to make such an assessment. The analyzed material contained 93 mite species obtained from 16,921 soil samples, which were collected between 1961 and 2017 in the whole area of Poland. The categories were assigned to species on the basis of the frequency of the species, but also other factors were taken into account, such as microhabitat specificity, vulnerability to detrimental conditions, and shrinking of local populations. One of the analyzed species can now be regarded as extinct, over 25% of the species (26 spp.) were labeled as critically endangered, and most of them (33 spp.) were categorized as vulnerable—the other species were assigned to the categories endangered (13 spp.), near threatened (10 spp.), and least concern (10 spp.).

## Introduction

The deteriorating condition of the natural environment—which is evident in the shrinkage of natural habitats, a decrease in population abundance of many species, and, as a result, an overall decline in biodiversity—is the major reason why so many species of the European fauna have been listed in the European Red List, which is part of the IUCN Red List of Threatened Species. Among the endangered species there are species from all groups of vertebrates. As for invertebrates, among the endangered species there are 44% of all fresh-water mollusks and 20% of some terrestrial mollusks, 15% of dragonflies, 11% of saproxylic beetles, and 9% of butterflies (European Commission 2017).

This clearly suggests that the assessment of the extent to which species are endangered focuses mainly on vertebrates and the invertebrate species that can be easily found in the examined area. There is virtually no research into the scale of extinction of microscopic arthropods, especially those inhabiting soil. It is impossible to observe such organisms directly in the examined area for a long period of time, which also means that it is impossible to determine the stability and extent of the changes occurring in populations of the respective species (Błoszyk 1999; Niedbała 2000; André et al. 2002). There is no doubt that this holds for soil mesofauna, and mites (Acari) are among them. Soil habitats, and other ecosystem components, are often polluted causing environmental degradation, which can have a tremendous impact on species composition and the abundance of mite communities in a given area (Kaczmarek and Seniczak 1994, 1998; Napierała 2008; Napierała et al. 2015b). What is more important, soil mites are pivotal in nutrient cycling, soil formation, and decomposition of organic matter, which in turn can affect soil fertility and plant growth, and therefore these organisms are also important for economic reasons (Jeffery and Gardi 2010).

Increasing anthropopressure and the subsequent soil contamination, soil erosion, salinization, physical degradation, and climate change are responsible for that fact that many species of soil fauna have become threatened (Jeffery and Gardi 2010). Moreover, it has already been proven that overall soil biodiversity is in decline (Jones et al. 2012). For this reason special attention is paid to soil biodiversity by the European Union, which can be seen in the EU Biodiversity Strategy until 2020 (European Commission 2011). The effort aimed at realization of the strategy will be continued in order to fill this void in the research, including the research into the mapping and assessing ecosystem services in Europe, which will help to learn more about the influence of climate changes on biodiversity, and the role of soil biodiversity in delivering key ecosystem services, such as carbon sequestration and food supply. The commitment of the EU to soil biodiversity protection has been further supported by the International Convention on Biological Diversity, where its importance as a key player in sustainable agriculture was strengthened during the 2010 conference of the parties to the convention in Nagoya, Japan (Jones et al. 2012).

The Red Lists of endangered species rarely contain any species of soil fauna, especially arachnids (Arachnida). The IUCN Red List of Threatened Species (2017) contains only 0.24% species from this class out of all 102,248 species described so far (Chapman 2009). Various species of spiders constitute the most numerous group of assessed species (199 spp. of Araneae, but also 21 spp. of Opiliones, and 13 spp. of false scorpions, Pseudoscorpiones) (Red List Category summary for all animal classes and orders; IUCN Red List of Threatened Species 2017). Only a few species from the other orders, such as whip spiders (Amblypygi), scorpions (Scorpiones), and short-tailed whip scorpions (Schizomida), have been classified as endangered (IUCN Red List of Threatened Species 2017). As for mites, there is only one species that has been classified as endangered (EN), namely the moss mite (Oribatida) *Scheloribates evanescens* Wallwork, which was classified in 2014 (IUCN Red List of Threatened Species 2017). There are only two species of soil invertebrates inhabiting Poland that have been included in the ‘Polish red data book of animals: invertebrates’ (Głowaciński and Nowacki 2004), i.e., one of the Opiliones (*Siro carpathicus*) and one of the Pseudoscorpiones (*Neobisium polonicum*), both described by Prof. Jan Rafalski from Bieszczady (SE Poland). These two species have been classified as EN, and since October 2014 they have been legally protected in Poland. In 2008 the third volume of *Fauna Polski* [The Fauna of Poland] (Bogdanowicz et al. 2008) was published, in which the authors provide a list of mite species occurring in Poland. However, the list gives no information about the conservation status of the enumerated species, though the list contains information which can be helpful to estimate whether a given species is common and how frequently it occurs.

The fact that mites are quite susceptible to changes caused by industry and agriculture, and all temperature and soil moisture fluctuations that they cause, makes these organisms perfect bioindicators of soil health (Moore et al. 1984; Błoszyk 1998a, b; Gardi et al. 2002; Migliorini et al. 2004; Aspetti et al. 2010). However, there are very few studies that present results of regular long-term quantitative research into soil fauna, which is essential to determine the trajectory of changes in populations of soil fauna (Malmström et al. 2009). The numerous acarological studies conducted in Poland over the last 50 + years are the only exception in this respect (Niedbała 1972, 1976, 1990; Błaszak 1974; Rajski 1967, 1969; Kaźmierski 1980; Niedbała et al. 1981; Błoszyk 1983, 1999; Michocka 1987; Kaliszewski and Sell 1990; Siuda 1993; Gabryś and Mąkol 1995; Gabryś 1996; Wiśniewski 1997; Mąkol 2005; Gwiazdowicz 2007). For this reason Poland is probably the only country in which distribution of mites has been analyzed so comprehensively.

Because mites from the suborder Uropodina have been already thoroughly described based on long-term quantitative research (Athias-Binche 1977a, b, c, 1981a, b, c, 1982a, b, 1983; Błoszyk 1983, 1984, 1999; Wiśniewski and Hirschmann 1993; Wiśniewski 1997; Mašán 2001; Błoszyk et al. 2003a), the current study presents the Red List of threatened species of soil mites in Poland, and an analysis of the IUCN criteria for this group of organisms. In 2011, Cardoso et al. (2011) made an attempt to adapt the IUCN criteria to classify invertebrates according to the conservation status. Cardoso et al. (2011) provided a critical review of the IUCN criteria focusing on the applicability of these criteria in establishing the conservation status of invertebrates, and they proposed how to effectively adapt the criteria. We decided to go a bit further and also analyze the IUCN criteria to see whether they can be helpful in estimating the conservation status of soil mesofauna, and used mites from the suborder Uropodina as a model group. Furthermore, this study also presents an assessment of the conservation status of Uropodina species in Poland, and a classification of the species according to the IUCN criteria. Analysis of the population abundance of the discussed species from this group and the assessment of their conservation status was carried out on the basis of direct observations plus data from previous studies.

### Mites from the suborder Uropodina as a model group

Cardoso et al. (2011) analyzed the IUCN criteria and the applicability of these criteria in establishing the conservation status of invertebrates, and they also suggested how to effectively modify these criteria. They too believe that most of these criteria, which are usually assigned on the basis of species abundance, are not applicable to invertebrates because there is no effective method of estimating abundance of populations of any terrestrial species of invertebrates in the natural environment (Kozlowski 2008; Cardoso et al. 2011). Regardless of the sampling method, the final results will always diverge from the actual abundance of the species (Niedbała 2000; André et al. 2002). This can be observed especially in the case of very small invertebrates which live in specific microhabitats, and they can form local populations within an area of 1 m^2^ (Napierała 2008). This applies with no doubt to mites (Acarina), as they are small arachnids inhabiting, e.g., soil and litter of forest ecosystems, as well as open environments and unstable microhabitats (such as bird nests, mammal nests, anthills, dead wood, and excrements of vertebrates). However, little has been done so far to establish the conservation status of species from this group of arachnids with its worldwide distribution. Many of these species were described on the basis of material collected only in one ground plot (does this mean high endemism?), sometimes with a low number of specimens, occasionally just a few (does this mean low abundance?). Moreover, many species were found only once and, despite intensive subsequent exploration, have never been recorded since. Does this mean that they have become extinct? Are the examined microhabitats the best places, and is the time the best to find these species? The answer to each of these questions can be proper. The major problem here is of course the size of mites, which makes direct observation impossible in any site. Thus, gaining information about the actual distribution and abundance of local populations is extremely hard and laborious. During the processes of collecting samples, extracting specimens, and until preparing microscopic slides it is unclear what species the collected samples contain. Research conducted so far focuses mainly on the description of new taxa, disregarding their biology, ecology, and zoogeography—for this reason the available accounts about these matters are rather obscure and fragmentary. Mites from the suborder Uropodina are among the exceptions in this respect (Athias-Binche 1977a, b, c, 1981a, b, c, 1982a, b, 1983; Błoszyk 1984, 1985, 1999; Wiśniewski and Hirschmann 1993; Wiśniewski 1997; Mašán 2001; Błoszyk et al. 2003a). These arachnids, with highly diversified morphology, have been the primary focus of interest for a long time for many acarologists, which is evident in the extensive research conducted so far. The data presented in earlier studies and our direct observations of these mites conducted for over 50 years allow the use of Uropodina as a model group to verify the IUCN criteria, and to propose certain modifications in relation to what is offered by Cardoso et al. (2011).

Mites from the suborder Uropodina are a well-known group in Europe. The number of European species that have been identified and described hitherto exceeds 440 (Wiśniewski and Hirschmann 1993). A rough estimate of the number of Uropodina species in Poland is 150 (Wiśniewski 1997) or 137 (Błoszyk 1999, 2008). One of the most specific characteristics of Uropodina is their great diversity in habitat preferences. The species living in soil and litter of forest ecosystems constitute over 60% of the Polish Uropodina fauna, whereas the other species (30%) inhabit unstable microhabitats, such as tree hollows, rotten tree trunks, anthills, bird and mammal nests, and animal feces, and about 9% occur in open habitats such as meadows, sandhills, xerophilous grasses etc. (see, e.g., Błoszyk 1999; Błoszyk et al. 2003a; Napierała and Błoszyk 2013).

The highest abundance of Uropodina occurs in places with a high percentage of organic matter such as litter of deciduous forests (frequency up to 10,000 specimens/m^2^) and dead wood and compost (Koehler 1997, 1999). Uropodina mites also have different trophic requirements. Many species are saprophagous, which means that they feed on dead organic matter from plants and animals (Karg 1993). Other species are mycetophagous, which feed on spores and mycelia (Faasch 1967; El-Banhawy et al. 1998). Apart from these, there are also predatory species, which hunt for nematodes, insect larvae, and oligochaetes (Faasch 1967; Ito 1971; El-Banhawy et al. 1998; Koehler 1997, 1999).

Their dispersal abilities and reproduction strategies depend on the habitat in which they live (e.g., Błoszyk 1999). It has been shown in many studies (e.g., Mašán 2001; Błoszyk et al. 2003a, 2004; Napierała and Błoszyk 2013; Napierała et al. 2016) that unstable microhabitats are usually populated by both males and females, whereas soil habitats are often dominated by parthenogenetic species, which are characterized by immense reduction of males in their populations (Norton et al. 1993; Błoszyk et al. 2004). Deutonymphs of some species, especially those inhabiting unstable merocenoses, have developed the ability of passive dispersion, by means of phoresy (Faasch 1967; Athias-Binche 1984, 1993, 1994). They can be carried by various groups of insects, e.g. by myriapods, as well as in fur mammals and bird feathers (Gwiazdowicz 2000; Bajerlein and Błoszyk 2004; Bajerlein et al. 2006; Napierała et al. 2015a).

Uropodina mites have also very specific habitat preferences. Looking at the discerned ecological elements of Poland one can say that most of Uropodina mites are stenobiotic and oligobiotic (70%), whereas only 6% are eurybiotic (Błoszyk 1999; Błoszyk et al. 2003a, 2004; Napierała 2008). Due to their narrow ecological tolerance Uropodina mites very quickly react to fluctuations in environmental conditions (both abiotic and biotic) by changes in species composition and abundance (Błoszyk 1999; Napierała 2008; Napierała et al. 2015b). For this reason mites from this group can be used as bioindicators of changes in the soil environment and, of course, they can be used to evaluate soil quality.

## Materials and methods

**Fig. 1:**
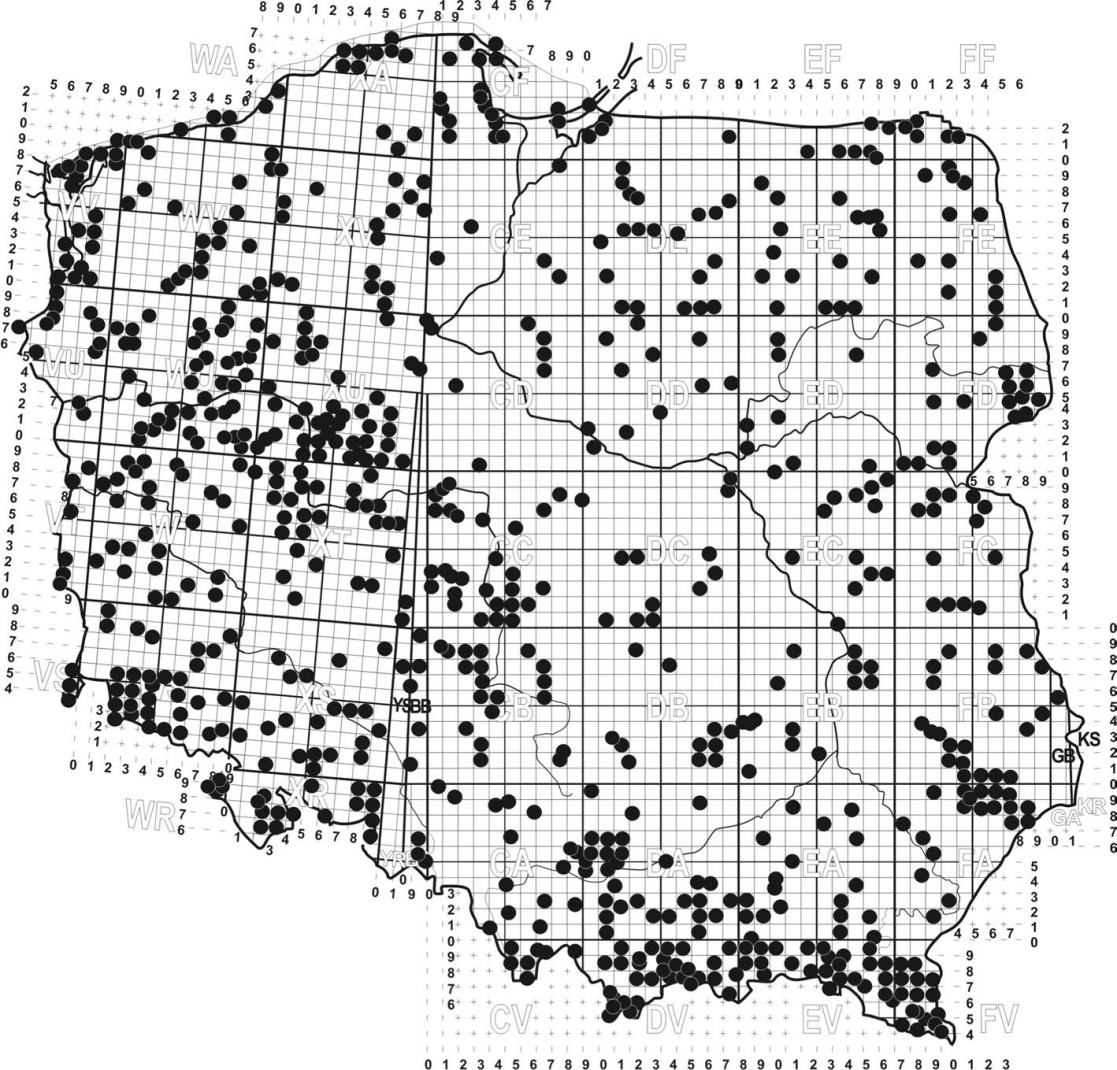
Areas of Poland where the material for the analysis was collected

The material for the analysis comprises 16,921 soil samples collected between 1961 and 2017 regardless of the season, by different researchers, in the whole area of Poland. Out of all soil samples 39% (6599) were qualitative samples—sieved litter and soil, as well as non-sieved samples of dead wood (with 0.5–1 L volume), and 61% (10,322) were quantitative samples with the size between 16 and 100 cm^2^. The quantitative samples were collected with a metal frame of 4 × 4 cm, or with a cylinder at the depth of up to 10 cm (Błoszyk 1999). The material was harvested from three types of environment, i.e., open, forest, and merocoenoses. The samples were collected evenly in the whole area of Poland (Fig. 1). This method allows to estimate the approximate distribution of species in the examined areas inhabited by local populations (Fig. 1) (Błoszyk 1999). These samples contained 93 species of Uropodina, which were represented by 158,051specimens.

Our analysis is also based on the data stored in the digital database of *Natural History Collections* at the Faculty of Biology at Adam Mickiewicz University in Poznań, and data from earlier studies.

### Criteria for classification of threatened species of mites from the suborder Uropodina according to the IUCN categories

This article employs the terms of categories proposed by the IUCN (2001, Categories and Criteria, version 3.1). Moreover, the paper also is a critical evaluation of the IUCN criteria used for establishing conservation status in terms of their usage to evaluate the status for mites from the suborder Uropodina. The new criteria proposed here may be used also for other groups of soil fauna organisms.

On the basis of our long-term research and the evidence available in the literature (Błoszyk 1983, 1999; Wiśniewski and Hirschmann 1993; Mašán 2001; Błoszyk et al. 2003a; Napierała and Błoszyk 2013), we claim that the parameters of dominance (D), frequency (F), and ecological importance (Q) are the most significant criteria in the evaluation of the conservation status of mites from the suborder Uropodina (see Kasprzak and Niedbała 1981). In the current study we have estimated the conservation status for each of the 93 Uropodina species listed in the study.

The analysis of the abundance and occurrence frequency of species in the samples employs dominance (D) and frequency (F) as biocenotic indices (Table 1). Moreover, the analysis also takes into account the synthetic index Q = √ D*F, where ‘Q’ stands for the index of ecological importance (Kasprzak and Niedbała 1981).

**Table 1:** Evaluation of frequency and abundance of species calculated for the dominance index (D) and frequency index (F)

Dominance	Frequency			
	Very frequent	Frequent	Rare	Sporadic
Abundant	D5–D4; F5–F4;	D5–D4; F3	D5–D4; F2	D5–D4; F1
Numerous	D3; F5–F4;	D3; F3;	D3; F2	D3; F1
Few	D2; F5–F4;	D2; F3	D2; F2	D2; F1
Very few	D1; F5–F4;	D1; F3	D1; F2	D1; F1

The adopted categories of zoocenological indices (after Kasprzak and Niedbała 1981) are:

**Table:** 

Frequency (F)			Dominance (D)			Ecological importance (Q)		
F5	Euconstants	> 30%	D5	Eudominants	> 10%	Q5	Very frequent	> 38.73
F4	Constants	15.1–30%	D4	Dominants	5.1–10%	Q4	Frequent	21.32–38.73
F3	Subconstants	7.1–15%	D3	Subdominants	2.1–5%	Q3	Rare	10.35–21.21
F2	Accessory species	3–7%	D2	Residents	1.1–2%	Q2	Sporadic	3.87–10.25
F1	Accidents	< 3%	D1	Subresidents	< 1%	Q1	Very sporadic	< 3.87

Also other factors were taken into account, for example, the fact that a species lives in a specific microhabitat, its ecological vulnerability (e.g., colonization of unique habitats), and the shrinking of local populations observed by the authors during the research period. The analysis also takes into consideration whether a given species is common or rare, the extent to which the species is threatened, and the pace of shrinking of the environment inhabited by the species, as these factors can have a direct impact on occurrence of Uropodina mites in such places. Among such habitats there are, for example, shrinking wetland areas, xerothermic grasslands, old forests (> 120 years old), tree hollows, nests of some species of birds, etc. The species associated with such habitats have been classified with the lowest value of the ‘habitat’ parameter (H). Additional parameters are geographic range (Gr), which can be broad or restricted, the dynamics of geographic range (Dgr), which can be stable or shrinking, and population reduction (Pr), which shows the potential reduction or increase in the abundance of local populations of a species. These parameters had the following values:

**Table:** 

Habitat (H)		Geographic range (Gr)		Dynamics of geographic range (DGr)		Population reduction (Pr)		
4	Eurytop	4	Very broad	3	Extending	Pr1	Population abundance very low or with decreasing tendency	− 2
3	Politop	3	Broad	2	Stable	Pr2	Stable population abundance or with increasing tendency	2
2	Oligotop	2	Restricted	1	Shrinking			
1	Stenotop	1	Endemic					

Sources: (H) Błoszyk et al. (2004), Napierała and Błoszyk (2013), (Gr) Wiśniewski and Hirschmann (1993), Błoszyk (1999), Mašán (2001), Błoszyk et al. (2003a); unpublished data, (DGr) Wiśniewski and Hirschmann (1993), Błoszyk (1999), Mašán (2001), Błoszyk et al. (2003a), unpublished data

The sum of these parameters stands for the value of the Endangered Index (EnI), which was used to establish the categories of threatening for the listed Uropodina species: EnI = F + D + Q + H + Gr + DGr + Pr.

### Critical overview of the IUCN criteria in evaluation of conservation status of soil mesofauna

The following sub-sections show how to adapt the IUCN categories and criteria in evaluation of the conservation status of soil mites, with examples of Uropodina mites found in Poland.

#### Criterion A: population reduction

To evaluate the reduction of mite populations it is possible to use a simple index of constancy (especially in quantitative analyses) or to measure the frequency of mite specimens in samples (in qualitative analyses). Decrease in the frequency of occurrence at a local scale in long-term studies (e.g., a national park or nature reserve), at a macro-scale (any administrative unit or country), or globally (in the whole area of a continent or geographical region) means that the existence of the species is threatened. A relegation of a species from category F5 (euconstants; frequency/constancy in samples > 50%) (frequency indexes after Błoszyk 1999) and F4 (constants; frequency/constancy in samples 30.1–50%) to F3 (subconstants; frequency/constancy in samples 15.1–30%) allows to label it as ‘vulnerable’ (VU). This means a considerable decrease in the number of the local populations in the natural environment. The decrease of this zoocenological parameter to F2 (accessory species; frequency/constancy in samples 5.1–15%) allows to assign the species to the category ‘endangered’ (EN), and when it can be given F1 (accidents; frequency/constancy in samples 5.1–15%) the species has the status of ‘critically endangered’ (CR). If a species occurs in samples at F2 or F1, it is usually stenotopic or oligotopic (this is evident in the habitat preferences of the species), which means that such species are very vulnerable to any detrimental change in environmental conditions.

#### Criterion B: geographic range

It is hard to establish the exact area of occurrence of invertebrates in the natural environment (Niedbała 2000; André et al. 2002; Lewis and Senior 2011). In the case of soil mites it depends on the extent to which a given area has been examined. The range of occurrence in a given area is determined by the most distant ground plots. In the case of data from other studies than our own, a ground plot can be considered reliable if a large number of specimens was found in it during one collecting session, or if the session is repeated at least twice. Single specimens of a species found beyond the range of occurrence or synanthropic environments (such as parks, agrocenoses, and urbanized areas) should always be considered with great caution. It is possible that the specimens of a given species found in such places had been introduced there by humans, and it is very unlikely that a population will survive in that place. Monographs and catalogues can be valuable sources of information about distribution of species of invertebrates (usually in the area of a country). As for Poland, *Monografie Fauny Polski* [Monographs of Polish Fauna] and *Katalog Fauny Polski* [Catalogue of Polish Fauna] seem to be reliable sources of information. Volumes that provide information about the exact locations where specimens were collected or that contain maps with the distribution of described species are extremely valuable (Błaszak 1974; Niedbała 1976; Błoszyk 1999). Lists of species compiled for a given country, usually with no information about the abundance, frequency of occurrence, and distribution, are of course far less informative (Hirschmann and Hutu 1974; Hirschmann 1979; Wiśniewski and Hirschmann 1993). Nowadays the global positioning system (GPS) allows to localize precisely the places of sample collection, and computer software, as well as GPS systems, can visualize the distribution of whole local populations, or even single specimens (Błoszyk et al. 2013). Thus, it is possible to establish precisely the area and range of occurrence of a species. This in turn means that regular monitoring of the range of occurrence of a species can tell whether it remains within the range, it is retrieving from a place colonized earlier, or is expanding. As it is impossible to establish the exact boundaries of the range for invertebrates, we claim that in the case of soil fauna (especially soil mites) the range becomes narrower or broader when during a period of 10 years the most distant ground plots (which mark the range) for a given species retracted or move further for at least 20 km, which is twice the basic unit of the UTM grid (10 × 10 km).

#### Criterion C: small population size and decline

Cardoso et al. (2011) claim that the current abundance limits for populations of invertebrates proposed by IUCN are not reasonable because the values are too low and irrelevant to the abundance of invertebrates, and the use of these values can lead to underestimation of the extinction threat—the claim seems to be valid. Even a very high abundance of a species does not protect it against extinction. This is true for both vertebrates (e.g., the passenger pigeon) and invertebrates (e.g., ants, termites, wasps) (Dorst and Sikora 1971), as it is quite easy to cause extinction of a large local population. In the case of mites, for example, abundance of mites in dead wood can be very high, but if the wood is removed from the forest, the species will be taken away with the wood, as well as many other saproxylic and cortical invertebrates (Stokland et al. 2012). Other examples are species inhabiting bird nests, small mammals, or excrements of big vertebrates (see e.g. Napierała and Błoszyk 2013). Thus, regardless of the current abundance, species that live in specific unstable and isolated microhabitats should be also regarded as threatened.

#### Criterion D: very small or restricted population

Assuming that it would be possible to estimate their actual abundance, for mites it would be insufficient to accept the current species abundance limits: ‘critically endangered if n (number of specimens) < 50, endangered if n < 250, and vulnerable if n < 1000, or AOO (area of occupancy) < 20 km^2^ or ≤ 5 locations‘—these limits would lead to underestimation of the conservation status. For example, *Metagynella paradoxa* has been found only twice in Poland in over 2000 samples of dead wood and wood galleries under tree bark (Błoszyk, unpubl. data), but in each case the number of specimens was quite large (> 50 and > 250). This species would seem to be extremely threatened due to the low number of places where it has been found so far. Moreover, the habitat of this species is exposed to destruction by felling old trees and these very rare populations could have been destroyed.

#### Criterion E: quantitative analysis of extinction risk

As has been pointed out by Cardoso et al. (2011), the criterion ‘Quantitative analysis of extinction risk’ should also take into account the possibility of habitat destruction. This is important especially in the case of stenotopic species, which occur mainly in unstable microhabitats, or habitats that are liable to frequent transformations (e.g., due to human activity). Many species of mites have very specific preferences for only one type of unstable microhabitat, and for this reason they often occur only in this particular type of microhabitat, where they form communities, very unique in their species composition and dominance structure (Napierała and Błoszyk 2013). The occurrence of such species in forest ecosystems increases the biodiversity of Uropodina in such communities by one-third (Błoszyk 1999; Błoszyk et al. 2003b; Napierała and Błoszyk 2013). On the other hand, cutting down 10 ha of one of the largest remaining parts of the primeval forest in Puszcza Białowieska (NE Poland) eliminates most of the litter species of mites from the suborder Uropodina, and reconstruction of such as community in a hornbeam forest would take roughly 80–120 years (Błoszyk unpubl. data). Furthermore, the IUCN criteria do not take into consideration the fact that estimating the probability of extinction of a particular number of specimens in a given period of time is extremely hard (Akçakaya et al. 2006). It should be borne in mind that abundance of invertebrates can fluctuate considerably (daily, seasonally, and annually). For this reason it is impossible to state definitely whether changes in abundance observed at a given time are not a reaction to changes in environmental conditions. In the case of invertebrates, frequency of occurrence of a species in a certain environment seems to be a much better criterion.

Based on the criteria adduced above, we present an evaluation of the conservation status for Uropodina species found in Poland. This is in fact a first Red List for mites inhabiting central Europe. Our evaluation embraces the whole area of Poland.

## Results and discussion

### The IUCN classification of mites from the suborder Uropodina

**Fig. 2:**
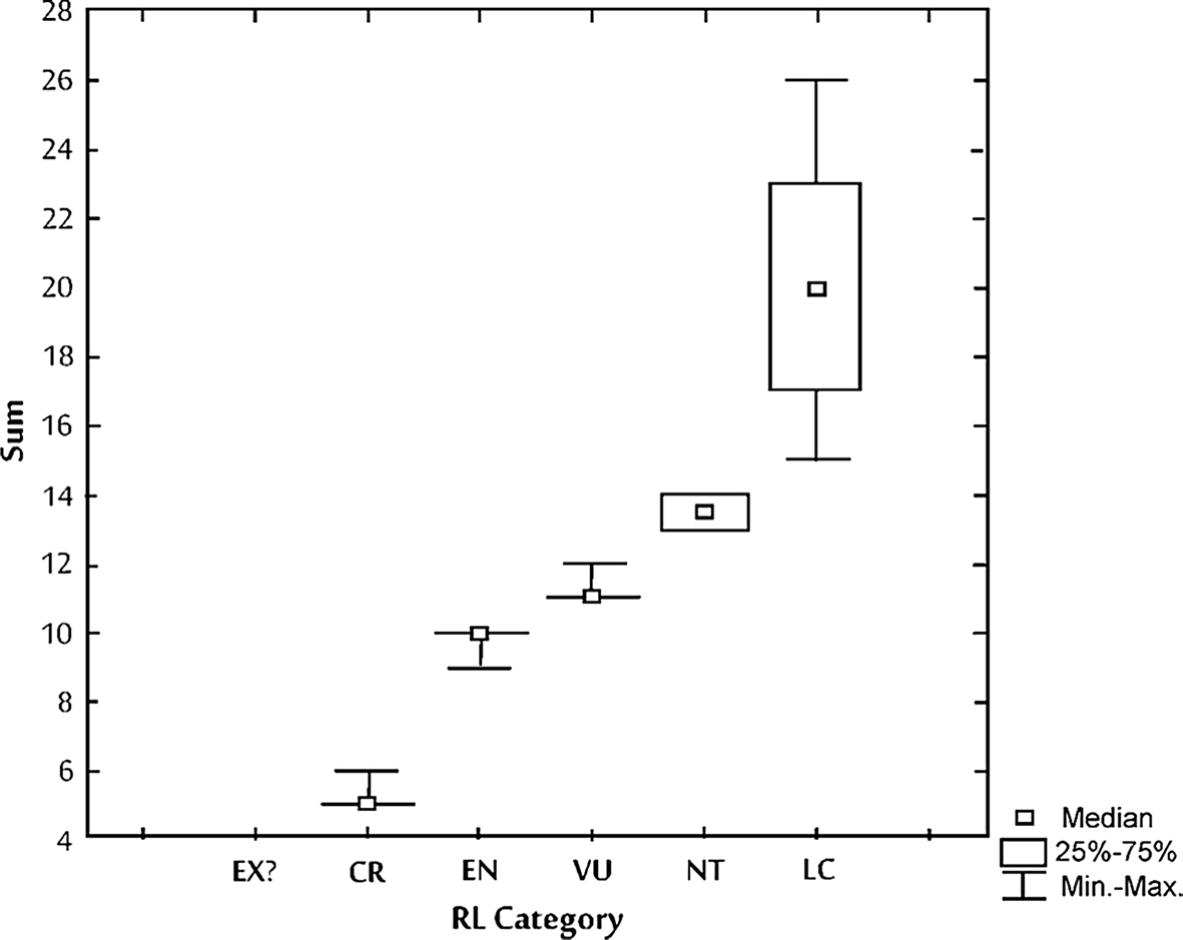
Values of EnI index converted into IUCN categories: EX (extinct) = EnI < 9 and species has not been recorded for at least 65 years; CR (critically endangered) = EnI < 9; EN (endangered) = EnI 9–10; VU (vulnerable) = EnI 11–12; NT (near threatened) = EnI 13–14; LC (least concern) = EnI > 14

The current study offers new criteria of classification for soil fauna, resting on nomenclature of the IUCN categories. The EnI index calculated for all Uropodina species listed in the study fluctuated between 5 and 26 (Table 2). The analysis of the EnI values allowed to distinguish different classes of this index, which were later converted to the IUCN criteria (Table 2, Fig. 2).

**Table 2:** Number of species (N) and their share (%) in different classes of EnI index (see Fig. 2 for explanation of the Red List categories)

RL category	Sum	5	6	9	10	11	12	13	14	15	17	19	20	21	23	24	26	Total
EX?	N	1	0	0	0	0	0	0	0	0	0	0	0	0	0	0	0	1
	%	100	0	0	0	0	0	0	0	0	0	0	0	0	0	0	0	
CR	N	23	3	0	0	0	0	0	0	0	0	0	0	0	0	0	0	26
	%	88	12	0	0	0	0	0	0	0	0	0	0	0	0	0	0	
EN	N	0	0	1	12	0	0	0	0	0	0	0	0	0	0	0	0	13
	%	0	0	8	92	0	0	0	0	0	0	0	0	0	0	0	0	
VU	N	0	0	0	0	25	8	0	0	0	0	0	0	0	0	0	0	33
	%	0	0	0	0	76	24	0	0	0	0	0	0	0	0	0	0	
NT	N	0	0	0	0	0	0	5	5	0	0	0	0	0	0	0	0	10
	%	0	0	0	0	0	0	50	50	0	0	0	0	0	0	0	0	
LC	N	0	0	0	0	0	0	0	0	2	1	1	2	1	1	1	1	10
	%	0	0	0	0	0	0	0	0	20	10	10	20	10	10	10	10	
Total	N	24	3	1	12	25	8	5	5	2	1	1	2	1	1	1	1	93

#### Evaluation of abundance and frequency of selected species

Out of the 93 species of Uropodina found in Poland only four can be regarded as common (Tables 3, 4). These are *Trachytes aegrota*, *Olodiscus minima*, *Oodinychus ovalis* and *Urodiaspis tecta*, which are eurytopic or polytopic species (Błoszyk 1999). *Trachytes irenae* and *Oodinychus karawaiewi* were also abundant, though they were found in fewer locations—*T. irenae* occurs only in the south of Poland, where the species has its northern range, whereas *O. karawaiewi* occurs mainly in areas with strong anthropopressure (Błoszyk 1999; Błoszyk et al. 2006a, b). None of the species was exceptionally abundant and rare at the same time. *Trachytes pauperior*, which has very specific preferences for soil moisture, was abundant and frequent in the whole area of Poland, as is *Dinychus perforatus*, which also has very specific preferences for soil moisture, though occurs far less frequently—both species occur mainly in litter and soil of various forest ecosystems (Błoszyk 1999).

**Table 3:** Zoocenological analysis of Uropodina species found in Poland

Dominance	Frequency			
	Very frequent	Frequent	Rare	Very rare
Very abundant	*T. aegrota*, *O. minima*	*O. ovalis*, *U. tecta*	*T. irenae*, *O. karawaiewi*	None
Abundant	None	*T. pauperior*	*D. perforatus*	*J. pulchella*, *L. orbicularis*, *P. borealis*
Few	None	None	*U. pannonica*	*J. pyriformis*, *N. splendida*, *A. infirmus*, *N. breviunguiculata*, *D. carinatus*
Very few	None	None	None	The other 76 species

**Table 4:** Evaluation of conservation status of Uropodina species in Poland on the basis of collected data and evidence from literature

Species	N	D	F	Q	H	Gr	DGr	Pr	EnI	RL category
*Trichouropoda barbatula* Willmann, 1950	1	D1	F1	Q1	1	2	1	− 2	5	EX?
*Oplitis alophora* (Berlese, 1903)	10	D1	F1	Q1	1	2	1	− 2	5	CR
*Oplitis franzi* Hirschmann et Zirngiebl-Nicol, 1969	1	D1	F1	Q1	1	2	1	− 2	5	CR
*Oplitis philocenta* (Trouessart, 1902)	1	D1	F1	Q1	1	2	1	− 2	5	CR
*Oplitis schmitzi* (Kneissl, 1908)	1	D1	F1	Q1	1	2	1	− 2	5	CR
*Oplitis stammeri* Hirschmann et Zirngiebl-Nicol, 1961	1	D1	F1	Q1	1	2	1	− 2	5	CR
*Oplitis wasmanni* (Kneissl, 1907)	1	D1	F1	Q1	1	2	1	− 2	5	CR
*Phaulodinychus copridis* (Oudemans, 1916)	1	D1	F1	Q1	1	2	1	− 2	5	CR
*Phaulodinychus spinosula* (Kneissl, 1916)	2	D1	F1	Q1	1	2	1	− 2	5	CR
*Polyaspinus schweizeri* Hutu, 1976	29	D1	F1	Q1	1	2	1	− 2	5	CR
*Trachyuropoda poppi* Hirschmann et Zirngiebl-Nicol, 1969	1	D1	F1	Q1	1	2	1	− 2	5	CR
*Trachyuropoda wasmanniana* Berlese, 1903	1	D1	F1	Q1	1	2	1	− 2	5	CR
*Trachyuropoda willmanni* Hirschmann et Zirngiebl-Nicol, 1969	17	D1	F1	Q1	1	2	1	− 2	5	CR
*Trichouropoda bipilis* (Vitzthum, 1921)	1	D1	F1	Q1	1	2	1	− 2	5	CR
*Trichouropoda dalarnaensis* Hirschmann et Zirngiebl-Nicol, 1961	2	D1	F1	Q1	1	2	1	− 2	5	CR
*Trichouropoda dialveolata* Hirschmann et Zirngiebl-Nicol, 1961	1	D1	F1	Q1	1	2	1	− 2	5	CR
*Trichouropoda longiovalis* Hirschmann et Zirngiebl-Nicol, 1961	1	D1	F1	Q1	1	2	1	− 2	5	CR
*Trichouropoda patavina* (G. Canestrini, 1885)	1	D1	F1	Q1	1	2	1	− 2	5	CR
*Uroobovella ipidis* (Vitzthum, 1923)	1	D1	F1	Q1	1	2	1	− 2	5	CR
*Uropoda italica* Hirschmann et Zirngiebl-Nicol, 1969	4	D1	F1	Q1	1	2	1	− 2	5	CR
*Uropoda undulata* Hirschmann et Zirngiebl-Nicol, 1969	38	D1	F1	Q1	1	2	1	− 2	5	CR
*Uropolyaspis hamulifera* Berlese, 1904*	25	D1	F1	Q1	1	2	1	− 2	5	CR
*Uroseius geieri* (Schweizer, 1961)	8	D1	F1	Q1	1	1	2	− 2	5	CR
*Uroseius hunzikeri* Schweizer, 1922	2	D1	F1	Q1	1	2	1	− 2	5	CR
*Protodinychus punctatus* Evans, 1957	2	D1	F1	Q1	1	2	2	− 2	6	CR
*Trachytes lamda* Berlese, 1903	440	D1	F1	Q1	2	2	1	− 2	6	CR
*Trachytes splendida* Hutu, 1973	12	D1	F1	Q1	1	2	2	− 2	6	CR
*Metagynella paradoxa* Berlese, 1919	172	D1	F1	Q1	1	2	1	2	9	EN
*Apionoseius infirmus* Berlese, 1887*	1762	D2	F1	Q1	1	2	2	2	10	EN
*Cilliba rafalskii* (Błoszyk, Stachowiak et Halliday, 2008)	621	D1	F1	Q1	2	1	2	2	10	EN
*Dinychus inermis* (C. L. Koch, 1841)	586	D1	F1	Q1	2	2	1	2	10	EN
*Discourella baloghi* Hirschmann et Zirngiebl-Nicol, 1969	998	D1	F1	Q1	2	2	1	2	10	EN
*Janetiella pyriformis* (Berlese, 1920)*	2633	D2	F1	Q1	1	2	2	2	10	EN
*Olodiscus kargi* (Hirschamann et Zirngiebl-Nicol, 1969)	257	D1	F1	Q1	2	2	1	2	10	EN
*Oodinychus spatulifera* (Moniez, 1892)	796	D1	F1	Q1	1	2	2	2	10	EN
*Oplitis minutissima* (Berlese, 1903)	8	D1	F1	Q1	1	2	2	2	10	EN
*Phaulodiaspis advena* (Trägårdh, 1922)	1063	D1	F1	Q1	1	2	2	2	10	EN
*Phaulodiaspis rackei* (Oudemans, 1912)	1483	D1	F1	Q1	1	2	2	2	10	EN
*Trachytes minima* Trägårdh, 1910	514	D1	F1	Q1	2	2	1	2	10	EN
*Trachyuropoda coccinea* (Michael, 1891)	152	D1	F1	Q1	1	2	2	2	10	EN
*Allodinychus flagelliger* (Berlese, 1910)	299	D1	F1	Q1	2	2	2	2	11	VU
*Cilliba selnicki* (Hirschmann et Zirngiebl-Nicol, 1969)	73	D1	F1	Q1	2	2	2	2	11	VU
*Dinychus woelkiei* Hirschmann et Zirngiebl-Nicol, 1969	833	D1	F1	Q1	2	2	2	2	11	VU
*Fuscouropoda appendiculata* (Berlese, 1910)	8	D1	F1	Q1	2	2	2	2	11	VU
*Iphiduropoda penicillata* (Hirschmann et Zirngiebl-Nicol, 1961)	76	D1	F1	Q1	2	2	2	2	11	VU
*Nenteria floralis* Karg, 1986	2	D1	F1	Q1	2	2	2	2	11	VU
*Nenteria pandioni* Wiśniewski et Hirschmann, 1985*	25	D1	F1	Q1	2	2	2	2	11	VU
*Oodinychus obscurasimilis* (Hirschmann et Zirngiebl-Nicol, 1961)	458	D1	F1	Q1	2	2	2	2	11	VU
*Polyaspis patavinus* Berlese, 1881	341	D1	F1	Q1	2	2	2	2	11	VU
*Polyaspis sansonei* Berlese, 1916	167	D1	F1	Q1	2	2	2	2	11	VU
*Trachytes montana* Willmann, 1953	23	D1	F1	Q1	2	2	2	2	11	VU
*Trematurella elegans* (Kramer, 1882)	756	D1	F1	Q1	2	2	2	2	11	VU
*Trichouropoda calcarata* (Hirschmann et Zirngiebl-Nicol, 1961)	56	D1	F1	Q1	2	2	2	2	11	VU
*Trichouropoda obscura* (C.L. Koch, 1836)	6	D1	F1	Q1	2	2	2	2	11	VU
*Trichouropoda polytricha* (Vitzthum, 1923)*	1000	D1	F1	Q1	1	3	2	2	11	VU
*Trichouropoda sociata* (Vitzthum, 1923)	1	D1	F1	Q1	2	2	2	2	11	VU
*Trichouropoda structura* (Hirschmann et Zirngiebl-Nicol, 1961)*	5	D1	F1	Q1	2	2	2	2	11	VU
*Trichouropoda tuberosa* (Hirschmann et Zirngiebl-Nicol, 1961)*	14	D1	F1	Q1	2	2	2	2	11	VU
*Uroobovella fimicola* (Berlese, 1903)	4	D1	F1	Q1	2	2	2	2	11	VU
*Uroobovella fracta* (Berlese, 1916)	4	D1	F1	Q1	2	2	2	2	11	VU
*Uroobovella nova* (Oudemans, 1902)	11	D1	F1	Q1	2	2	2	2	11	VU
*Uroobovella vinicolora* (Vitzthum, 1926)	2	D1	F1	Q1	2	2	2	2	11	VU
*Uroplitella conspicua* Berlese, 1903	22	D1	F1	Q1	2	2	2	2	11	VU
*Uroplitella paradoxa* (Canestrini et Berlese, 1884)	24	D1	F1	Q1	2	2	2	2	11	VU
*Urotrachytes formicarius* (Lubbock, 1881)	23	D1	F1	Q1	2	2	2	2	11	VU
*Dinychura cordieri* (Berlese, 1916)	531	D1	F1	Q1	2	2	3	2	12	VU
*Dinychus arcuatus* (Trägårdh, 1922)	482	D1	F1	Q1	3	2	2	2	12	VU
*Discourella modesta* (Leonardi, 1889)	368	D1	F1	Q1	2	3	2	2	12	VU
*Nenteria stylifera* (Berlese, 1904)	54	D1	F1	Q1	2	3	2	2	12	VU
*Olodiscus misella* (Berlese, 1916)	796	D1	F1	Q1	2	3	2	2	12	VU
*Urodiaspis stammeri* Hirschmann et Zirngiebl-Nicol, 1969	219	D1	F1	Q1	3	2	2	2	12	VU
*Uroobovella marginata* (C. L. Koch, 1829)	33	D1	F1	Q1	2	3	2	2	12	VU
*Uroobovella obovata* (Canestrini et Berlese, 1884)*	395	D1	F1	Q1	2	3	2	2	12	VU
*Cilliba cassideasimilis* (Błoszyk, Stachowiak et Halliday, 2008)	1492	D1	F1	Q1	3	3	2	2	13	NT
*Cilliba erlangensis* (Hirschmann et Zirngiebl-Nicol, 1969)	104	D1	F1	Q1	3	3	2	2	13	NT
*Dinychus carinatus* Berlese, 1903	1611	D2	F1	Q1	2	3	2	2	13	NT
*Leiodinychus orbicularis* (C. L. Koch, 1839)*	3358	D3	F1	Q1	1	3	2	2	13	NT
*Phaulodiaspis borealis* Sellnick, 1940*	3229	D3	F1	Q1	1	2	3	2	13	NT
*Cilliba cassidea* (Herman, 1804)	212	D1	F1	Q1	3	4	2	2	14	NT
*Janetiella pulchella* (Berlese, 1904)*	5418	D3	F1	Q1	2	3	2	2	14	NT
*Nenteria breviunguiculata* (Willmann, 1949)*	1752	D2	F1	Q1	2	4	2	2	14	NT
*Polyaspinus cylindricus* Berlese, 1916	1421	D1	F1	Q1	3	4	2	2	14	NT
*Uropoda orbicularis* (Müller, 1776)*	617	D1	F1	Q1	3	4	2	2	14	NT
*Neodiscopoma splendida* (Kramer, 1882)	2449	D2	F1	Q1	3	4	2	2	15	LC
*Urodiaspis pannonica* Willmann, 1952	1963	D2	F2	Q1	3	3	2	2	15	LC
*Dinychus perforatus* Kramer, 1882	3443	D3	F2	Q1	3	4	2	2	17	LC
*Trachytes irenae* Pecina, 1970	11,432	D4	F2	Q2	4	3	2	2	19	LC
*Trachytes pauperior* (Berlese, 1914)	7771	D3	F3	Q2	4	4	2	2	20	LC
*Urodiaspis tecta* (Kramer, 1876)	9269	D4	F3	Q2	3	4	2	2	20	LC
*Oodinychus karawaiewi* (Berlese, 1903)*	9177	D4	F2	Q2	4	4	3	2	21	LC
*Oodinychus ovalis* (C. L. Koch, 1839)*	25,261	D5	F3	Q3	4	4	2	2	23	LC
*Olodiscus minima* (Kramer, 1882)	16,024	D5	F4	Q3	4	4	2	2	24	LC
*Trachytes aegrota* (C. L. Koch, 1841)	33,287	D5	F5	Q4	4	4	2	2	26	LC
Total	158,051									

*N* number of specimens, *D* dominance, *F* frequency in samples, *Q* Q index, *H* habitat, *Gr* geographic range, *DGr* dynamic of geographic range, *Pr* population reduction, *EnI* endangered index, *RL category* Red List categories of conservation status according to IUCN (see Fig. 2 for explanation of the Red List categories)

*Phoretic species

Another group comprises species which form abundant local populations, but they occur fairly rarely (Table 3). Among these species are *Janetiella pulchella*, *Leiodinychus orbicularis* and *Phaulodiaspis borealis*. All these species occur in unstable microhabitats: *J. pulchella* occurs in dead wood merocenoses, *L. orbicularis* in bird nests, and *P. borealis* in burrows of the common mole (*Talpa europaea*) (Błoszyk 1999; Błoszyk et al. 2003a, b, 2015; Bajerlein and Błoszyk 2004; Napierała and Błoszyk 2013; Napierała et al. 2016). *Urodiaspis pannonica*, which occurs mainly in forests, was sparse and apparently sporadic in the whole area of Poland (Table 3) (Błoszyk 1999). Species like *Janetiella pyriformis*, *Neodiscopoma splendida*, *Apionoseius infirmus*, *Nenteria breviunguiculata*, and *Dinychus carinatus* are even less frequent. Only *N. splendida* is a soil species with interesting disjunctive geographical distribution, with two separate populations in Poland, one in the north and one in the south (Błoszyk 1999; Błoszyk et al. 2003a). The other species from this group occur in unstable merocoenoses: *J. pyriformis* and *D. carinatus* occur in tree hollows and different types of dead wood (Błoszyk 1999; Błoszyk et al. 2003a, 2015; Napierała and Błoszyk 2013), whereas *A. infirmus* and *N. breviunguiculata* occur mainly in old nests of predatory birds, as well as white and black storks (Bajerlein et al. 2006; Błoszyk and Gwiazdowicz 2006; Błoszyk et al. 2006a, b). The other 76 species are very sporadic and there are usually very few specimens found. Some of these species have been found so far only in a few locations as single specimens (Table 3).

#### Evaluation of conservation status

Evaluation of the conservation status of the 93 species of Uropodina in Poland (Table 4) indicates that one of the evaluated species (*Trichouropoda barbatula*) has been classified as extinct (EX), 26 species (28%) have been classified as critically endangered (CR), 13 (14%) as endangered (EN), 33 (35%) as vulnerable (VU), 10 (11%) as near threatened (NT), and 10 (11%) as least concern (LC).

#### Threatening factors

There are many factors that cause decline in species diversity and population abundance of Uropodina species in Poland, most importantly those directly involved in the destruction of habitats. As most Uropodina mites are forestal species, which prefer litter and soil, especially that of old forests (Athias-Binche 1977a, b, c, 1979, 1981a, b, c, 1982a, b, 1983; Błoszyk 1999; Mašán 2001), the factors that cause detrimental changes leading to degradation of forest ecosystems are the main focus in this study. These include both changes within the area of a forest complex and factors responsible for shrinking and fragmentation of forests. Fragmentation divides forests into separate areas and leads to shrinking of natural habitats (Pullin 2005). One of the biological consequences of forest fragmentation is the loss of the forestal character of these areas, which also means that such areas become prone to anthropopressure (Pullin 2005). This in turn can cause decline or even entire loss of typically forestal stenotopic species. The most important consequence of habitat fragmentation at the population level is that a population of a given species is divided into smaller populations (i.e., local populations). Among the species which have become endangered due to forest fragmentation and degradation are soil mites from the genus *Trachytes*. The gradual decline of local populations consisting of species from this genus has been observed for the last 40 years (Napierała 2008; Napierała et al. 2015b). One of these species—*T. lamda*—has been classified as endangered (EN)—a rare species occurring in old (mainly deciduous) forests found so far only in a few distant locations in Poland. Another good example is *T. splendida*, which is now critically endangered (CR) due to the process of cutting down and shrinking of old forests. Since the second half of the 1980 s the decline of populations of another rare species inhabiting central Europe—*T. minima* (EN)—has also been observed (Błoszyk 1999). This species occurred in Poland in two separate populations, in the Carpathians and Sudetes (Błoszyk 1980, 1999). Despite very intensive research the species has not been found in the area of the foothills of the Sudetes since the beginning of the 1990s. It is likely that this species has already become extinct in this region of Poland. Acid rain and accumulation of cross-border pollution in this region are probably the major two reasons responsible for this extinction. In this period researchers also observed dying of the forests in the Izery Mountains and in the Karkonosze Mountains, which obviously must have had a detrimental impact on the soil fauna in this region (Błoszyk 1995a, b).

Forests consisting of old trees are rich in microhabitat types, especially those containing dead wood. Felling old trees and fragmentation of large forests results in reduction of the living space of species inhabiting unstable microhabitats. For this reason the second group of threatening factors for Uropodina comprises activities which lead to loss or dispersion of merocenoses, which is crucial for many species, as roughly 30% of the species in Poland have strong habitat preferences for unstable microhabitats (Błoszyk 1999). One of the characteristics of such species is that they are usually abundant, but they are dispersed and occur only in a certain type of merocenosis (Napierała and Błoszyk 2013). The decline of such microhabitats and their scattering make it extremely hard for these small invertebrates to move and find a new appropriate microhabitat, which of course leads to extinction of a population. Although Uropodina mites can transfer from one place to another by means of phoresy (Athias-Binche 1984, 1993, 1994; Faasch 1967; Bajerlein and Błoszyk 2004), and they can quickly colonize new microhabitats, they may not survive when there are very few places with the right habitat conditions or when such places are too far. A good example of a critically endangered (CR) species living in microhabitats is *Oplitis alophora.* It has been found in dead wood and tree hollows of old beeches in Roztocze (SE Poland). The species is very rare and sparse mainly because it occurs locally and the old beech forests are shrinking. A similar situation applies to *Iphiduropoda penicillata*, which is endangered due to extensive and uncontrolled deforestation of old forests. This species is rare and not abundant in its preferred habitats dead wood, tree hollows of old deciduous forests, and nests of predatory birds. A solution to this problem in Polish forestry will be a law that allows to leave dead wood in forests, which will increase the abundance and biodiversity of the species inhabiting dead wood. However, such solutions require a lot of time because Uropodina prefer old trees which are over 100 years old (Błoszyk 1999; Napierała 2008; Napierała et al. 2009).

Besides dead wood, Uropodina also live in other merocoenoses, for example, nests of birds and mammals, as well as anthills (Błoszyk 1999; Wiśniewski and Hirschmann 1993; Mašán 2001; Bajerlein et al. 2006; Błoszyk and Gwiazdowicz 2006; Błoszyk et al. 2006a, b; Napierała and Błoszyk 2013). The abundance and diversity of the mite species inhabiting such places is directly dependent on the abundance of the hosts; therefore, any decrease in the number of the host species entails a decrease in the fauna inhabiting the nest, including Uropodina mites. A good example showing this dependency is *A. flagelliger*, which is quite common in nests of the white stork (Błoszyk et al. 2005, 2006a, b; Błoszyk and Gwiazdowicz 2006). Nests of the white stork are fairly evenly distributed over the whole area of Poland (Kania 2006; Maluśkiewicz and Tomaszewski 2012; Pietrowiak 2012; Tobółka 2012), and at least 1/3 of them can be inhabited by this mite (Błoszyk and Gwiazdowicz 2006). During the last couple of years people in some countries started to hunt for migrating white stork, especially in Lebanon, which in turn has caused a decrease in the number of nests inhabited by this bird in Poland. This implies shrinkage of habitats for *A. flagelliger*, probably resulting in an overall decrease in the number of local populations, and thus in a decrease in the whole country. In this situation *A. flagelliger* should be classified as vulnerable (VU). Similarly, *Uroseius hunzikeri* inhabits unstable merocenoses, such as nests of birds, burrows of the common mole, nests of bumblebees (*Bombus* sp.), and soil. This species is usually very sparse and therefore can be classified as critically endangered (CR).

Species inhabiting open environments constitute the smallest group among the Uropodina. Open environments are very rare or exposed to degradation. *Trachyuropoda willmanni* and *T. poppi* are good examples—both species inhabit rare xerothermic plant communities and they both have been classified as critically endangered (CR). The decline of xerophilous grasses observed in the last couple of years is caused by the lack of grazing and grass harvesting, which accelerates the natural succession in such areas, and the growing shrubs and trees pose a serious threat to the existence of these species. Also other species are endangered due to the decline of open environments, for example, the hygrophilous species of Uropodina, such as *Uropoda undulata* and *D. inermis*. The former lives in peatlands, alder forests and marshy forests, where they form local populations with low abundance. The latter prefers damp meadows, alder forests and marshy forests (Błoszyk 1999). Although *D. inermis* is still very common, it may soon become endangered due to the gradual lowering of the groundwater level, land improvement in damp areas and the draining of swamps and peatlands.

All these threatening factors usually occur at the regional level and they affect most of the Uropodina species. However, soil fauna is also affected by global changes, e.g. climate changes (Napierała et al. 2010). For example, *Uroseius gaieri* is a postglacial relict which requires low temperature to survive (Błoszyk and Olszanowski 1984). The only population of this species has been found so far on a rock cliff in Szczeliniec. Due to its low abundance and local occurrence, and in light of global warming, this relic species is, with no doubt, endangered and has been assigned to the category CR.

## Conclusions

The IUCN Red List contains only one mite species, namely *Scheloribates evanescens*, which is a species from the order Oribatida (IUCN Red List of Threatened Species 2017). This species has been given the EN category on the basis of merely eight specimens found in 1967 in two locations on Saint Helena Island (IUCN Red List of Threatened Species 2017). The research into this species has never been repeated and, therefore, it is unknown whether the range of occurrence of the species is really restricted to this small area and whether the species is endemic. Moreover, the research seems questionable because it does not say whether the low number of specimens stems from the fact that the species is rare, or from collection in the least favorable season of natural annual fluctuations, or perhaps the location was not typical for the species. Similar doubts appear in the case of studies with accounts of species of mites found only in one location (e.g., Kontschán 2003). Any attempt at evaluating the conservation status of a species based on only few specimens throws the research into question, as the category EN given in the classification may be a result of poor sampling, and it does not necessarily show the actual conservation status.

For the current analysis we had at their disposal data and materials obtained during a long-term research: 16,921 samples collected for over 50 years and a lot of information about the biology and ecology of the analyzed group of mites. This article is in fact the first attempt to compile a Red List for soil mites from the suborder Uropodina inhabiting central Europe. Moreover, this work summarizes 50 + years of research into this group of mites in Poland in the context of species conservation. We tried to estimate the current state of Uropodina populations for the listed species on the basis of the IUCN criteria with certain modifications to adapt them to this group of organisms, and to highlight those species that may become endangered in the future. The large number of stenotopic and oligotopic species found in the analyzed material indicates that these mites are vulnerable to any detrimental changes in environmental conditions, which can pose threats to them (Błoszyk 1999; Błoszyk et al. 2003a; Napierała 2008; Napierała et al. 2015b).

The results can also have a broader and more practical application, which is not restricted only to establishing the conservation status of Uropodina species. As mites from this group can be used as bioindicators, Uropodina can be helpful in the monitoring of soil condition (Błoszyk 1998a, b, 1999; Czarnota and Błoszyk 1998; Błoszyk et al. 2003a; Napierała 2008; Napierała and Błoszyk 2013). The Convention on Biological Diversity, signed and ratified by Poland and other countries, imposes a formal obligation to work out strategies, plans, and programs that will focus on protection and monitoring of elements of biological diversity, and to identify processes which may have negative impact on the protection of biological diversity. This obligation forces researchers and institutions responsible for environmental protection to search for methods that will allow the use of faunistic elements for monitoring purposes and to evaluate the condition of the environment, in this case of the soil. The attempts to use Uropodina mites to estimate soil condition in Poland are plentiful, especially for protected areas such as national parks, nature reserves (Błoszyk 1998a, b; Błoszyk and Szymkowiak 1999; Błoszyk and Krysiak 2000; Błoszyk et al. 2002, 2010; Napierała 2008; Napierała et al. 2015b), and places that are extremely valuable from the natural point of view, for example, Puszcza Białowieska, or the largest concentration of yews (*Taxus baccata*) in Europe (Błoszyk and Olszanowski 1999; Błoszyk and Szymkowiak 1999; Błoszyk, unpubl. data).

Several interesting conclusions have been drawn from the research conducted in Wielkopolska (W Poland) (Napierała 2008). The results of this research show that 80% of all species of Uropodina in Wielkopolska occurred in nature reserves and in Wielkopolski National Park, and both these places constitute only 2% of the whole area of the region. The species listed in our Red List have been classified as critically endangered, and they occurred mainly in places legally protected and in areas that are valuable from the natural point of view (Błoszyk 1998a, b; Błoszyk and Olszanowski 1999; Błoszyk and Szymkowiak 1999; Błoszyk and Krysiak 2000; Błoszyk et al. 2002, 2010; Napierała 2008; Napierała et al. 2015b). The results also show that it is extremely important to protect habitats through establishing legally protected areas and rational management of natural resources in order to preserve diversity of soil fauna and other small invertebrates (Jones et al. 2012; Convention on Biological Diversity).

The attempt at compiling a Red List for one of the groups of soil mesofauna on the basis of the modified IUCN criteria is also a response to the statement made by Cardoso et al. (2011), who claim that there is a clear need to fill in the void between the number of invertebrate species whose conservation status has not been evaluated yet and the increasing number of new species of invertebrates described in the literature (15,000 every year) (Clarivate analytics ION, Index to Organism Names). In the case of mites the number of species described so far is roughly over 55,000 species (Krantz and Walter 2009), and the number is increasing. We are fully aware of the fact that the results of the research presented here are only a small contribution to the evaluation of the conservation status of soil mites, though such research seems important because it may encourage other researchers and experts to compile similar lists for other groups of invertebrates. The amount of available information on many groups of soil mesofauna may never be sufficient to legally protect these small organisms. However, evaluating the conservation status of at least some of them, especially those already described in the literature, by means of appropriate criteria, with certain modifications if necessary, can show that these small organisms, just like insects or vertebrates, also require legal protection, as they are important elements of many ecosystems. The only effective way of protecting them is by establishing legally protected areas which are most valuable from the natural point of view, regardless of their current legal status.
